# The bacterium *Wolbachia* exploits host innate immunity to establish a symbiotic relationship with the dengue vector mosquito *Aedes aegypti*

**DOI:** 10.1038/ismej.2017.174

**Published:** 2017-11-03

**Authors:** Xiaoling Pan, Andrew Pike, Deepak Joshi, Guowu Bian, Michael J McFadden, Peng Lu, Xiao Liang, Fengrui Zhang, Alexander S Raikhel, Zhiyong Xi

**Affiliations:** 1 Department of Microbiology and Molecular Genetics, Michigan State University, East Lansing, MI 48824, USA; 2 School of Medicine, Hunan Normal University, Changsha, Hunan 410013, China; 3 Department of Entomology and Institute for Integrative Molecular Biology, University of California, Riverside, CA 92521, USA; 4 Sun Yat-sen University—Michigan State University Joint Center of Vector Control for Tropical Diseases, Guangzhou, Guangdong 510080, China

## Abstract

A host’s immune system plays a central role in shaping the composition of the microbiota and, in return, resident microbes influence immune responses. Symbiotic associations of the maternally transmitted bacterium *Wolbachia* occur with a wide range of arthropods. It is, however, absent from the dengue and Zika vector mosquito *Aedes aegypti* in nature. When *Wolbachia* is artificially forced to form symbiosis with this new mosquito host, it boosts the basal immune response and enhances the mosquito’s resistance to pathogens, including dengue, Zika virus and malaria parasites. The mechanisms involved in establishing a symbiotic relationship between *Wolbachia* and *A. aegypti*, and the long-term outcomes of this interaction, are not well understood. Here, we have demonstrated that both the immune deficiency (IMD) and Toll pathways are activated by the *Wolbachia* strain *w*AlbB upon its introduction into *A. aegypti.* Silencing the Toll and IMD pathways via RNA interference reduces the *w*AlbB load. Notably, *w*AlbB induces peptidoglycan recognition protein (PGRP)-LE expression in the carcass of *A. aegypti*, and its silencing results in a reduction of symbiont load. Using transgenic mosquitoes with stage-specific induction of the IMD and Toll pathways, we have shown that elevated *w*AlbB infection in these mosquitoes is maintained via maternal transmission. These results indicate that host innate immunity is utilized to establish and promote host-microbial symbiosis. Our results will facilitate a long-term projection of the stability of the *Wolbachia*–*A. aegypti* mosquito system that is being developed to control dengue and Zika virus transmission to humans.

## Introduction

Mosquitoes transmit numerous devastating human diseases such as Zika, dengue and malaria. Both the Zika and dengue viruses are vectored primarily by the *Aedes aegypti* mosquito. While dengue fever has spread to five continents with over 3 billion people at risk of contracting the disease (Bhatt *et al.*, 2013), Zika transmission recently resulted in a public health emergency of international concern. Failing mosquito-control methods, as well as the lack of an anti-dengue vaccine and anti-viral drugs, have contributed to this situation. Recently, several non-chemical insecticide-based approaches have been developed for the control of mosquito populations, including the utilization of *Wolbachia* (Xi *et al.*, 2005; Bian *et al.*, 2010; McMeniman and O'Neill, 2010; Hoffmann *et al.*, 2011).

*Wolbachia spp*. are obligate, maternally inherited endosymbionts that infect >65% of all the insect species and ~28% of the surveyed mosquito species (Kittayapong *et al.*, 2000; Ricci *et al.*, 2002; Werren *et al.*, 2008). Through cytoplasmic incompatibility (CI), *Wolbachia* induce early embryonic death when a *Wolbachia*-infected male mates with a female that is either uninfected or infected by a different type of *Wolbachia* (Dobson, 2003). Cytoplasmic incompatibility provides a reproductive advantage to infected females over uninfected females, resulting in the invasion of *Wolbachia* into a population. *Wolbachia* can also interfere with pathogen infection and directly inhibit a variety of human pathogens including dengue virus, Zika virus, malaria parasites and filarial worms (Kambris *et al.*, 2009; Moreira *et al.*, 2009; Bian *et al.*, 2010, 2013; Dutra *et al.*, 2016). These features of *Wolbachia* have generated a great deal of interest in creating *Wolbachia*-based systems for diminishing the mosquito’s ability to transmit pathogens and/or suppress mosquito population (Xi *et al.*, 2005; Brelsfoard *et al.*, 2008; Hoffmann *et al.*, 2011; Bian *et al.*, 2013; Bourtzis *et al.*, 2014). However, the mechanisms responsible for establishing persistent symbiosis between this bacterium and new hosts have not been well understood, despite the importance of this question for predicting the long-term success of *Wolbachia*-based vector-borne disease control programs.

Insects, including mosquitoes, have developed a highly effective innate immune system to defend against harmful microbes they encounter during their life cycle. The immune response is initiated by identification of microbe-associated molecular patterns via pattern-recognition receptors (PRRs), which results in the activation of immune signaling pathways and expression of effector molecules to suppress infection (Dimopoulos, 2003; Waterhouse *et al.*, 2007). In mosquitoes, the Toll and immune deficiency (IMD) signaling pathways activate two distinct nuclear factor-kappaB-IkB transcription factors, REL1 and REL2, respectively, to induce the expression of antimicrobial peptides (Waterhouse *et al.*, 2007). This innate immunity has a significant role in determining the mosquito’s ability to transmit human pathogens, such as dengue virus and malaria parasites (Meister *et al.*, 2005; Dong *et al.*, 2006; Frolet *et al.*, 2006; Xi *et al.*, 2008b). While the Toll pathway functions to defend against Gram-positive bacteria, fungi, dengue virus and *Plasmodium berghei* (Bian *et al.*, 2005; Shin *et al.*, 2005; Frolet *et al.*, 2006; Xi *et al.*, 2008b), activation of the IMD pathway inhibits Gram-negative bacterium and *Plasmodium falciparum* (Meister *et al.*, 2005). Overall, such responses to pathogen invasion characterize inducible immunity. In contrast, constitutive immunity, also referred to as basal immunity, represents a surveillance mechanism that functions prior to pathogen invasion. Data have shown that basal immunity is influenced by the commensal microbiota within a mosquito (Frolet *et al.*, 2006). Removal of these resident microbes reduces the baseline level of immune effectors, resulting in increased susceptibility of the mosquito to both dengue virus and malaria parasites (Xi *et al.*, 2008b; Dong *et al.*, 2009). Recent studies have demonstrated that the symbiotic microbiota contributes to the buildup of host basal immunity and the function of certain immune responses in both vertebrates and invertebrates (Kumar *et al.*, 2010; Hooper *et al.*, 2012). For example, in multiple hosts, gut bacterial ligands from the microbiota signal through PRRs to promote the development of host tissues, including the immune system, and protect the host from diseases (Chu and Mazmanian, 2013).

Although *A. aegypti*, the primary dengue vector, does not carry *Wolbachia* in nature, the *Wolbachia w*AlbB strain has been successfully transferred from its native host, *Aedes albopictus,* into this mosquito species and, since then, the *w*AlbB-infected *A. aegypti* strain (WB1) has been maintained for >12 years in a laboratory population (Xi *et al.*, 2005). In contrast to *A. albopictus*, in which no impact of *Wolbachia* on immunity has been observed (Bourtzis *et al.*, 2000; Molloy and Sinkins, 2015), *w*AlbB induces the production of reactive oxygen species and activates the Toll pathway in WB1 strain *A. aegypti* (Pan *et al.*, 2012). In addition, *w*AlbB has been shown to grow to significantly higher densities in WB1 than in *A. albopictus* and invade different tissues such as the fat body, midgut, salivary glands, and ovaries (Lu *et al.*, 2012). In *A. albopictus*, the majority of *Wolbachia* reside in the ovaries (Lu *et al.*, 2012). Although it is known that *Wolbachia* infection results in immune activation in transfected *A. aegypti* lines (Kambris *et al.*, 2009; Moreira *et al.*, 2009; Bian *et al.*, 2010, 2013), it is unclear what role these immune responses have in establishing the symbiotic relationship of this bacterium and its new host. Specifically, it is unknown whether boosting the immune response prevents over-proliferation of *Wolbachia* or promotes the establishment and maintenance of symbiosis with this bacterium. Elucidating this information is essential for predicting the long-term persistence of the symbiotic relationship of *Wolbachia* with its new host, *A. aegypti*, and the potential success of the *Wolbachia-*mosquito system that is presently being used to control mosquito populations.

In this work, we utilized reverse genetic and transgenic tools to manipulate the mosquito innate immune system and test its influence on the interaction between *w*AlbB and *A. aegypti*. Suppression of either the IMD pathway alone or both the Toll and IMD pathways reduced the *Wolbachia* load, whereas activation of both pathways increased *Wolbachia* load, indicating the importance of innate immunity in fostering the symbiotic relationship between *w*AlbB and *A. aegypti*. Using transgenic mosquitoes with stage-specific induction of the IMD and Toll pathways, we have shown that the elevated *w*AlbB infection in this background is maintained via maternal transmission, which is a hallmark of an established symbiotic relationship. Overall, our results suggest that *Wolbachia* utilizes the host immune system to promote its symbiotic relationship with *A. aegypti* mosquitoes.

## Results

### *Wolbachia w*AlbB activates both the Toll and IMD pathways and protects *A. aegypti* against bacteria and fungi

**Figure 1:**
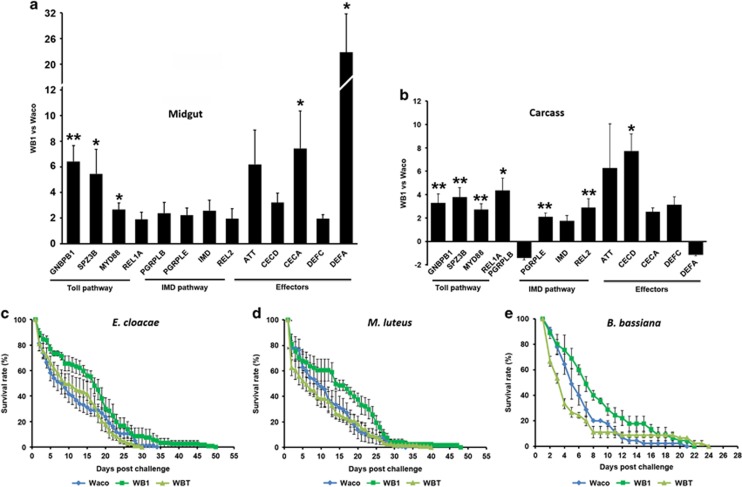
*w*AlbB induces both the IMD and Toll pathways in the *A. aegypti* line WB1. The fold change in the expression of Toll and IMD pathway genes, measured by qRT-PCR, in the midgut (**a**) and remaining carcass (**b**) of *w*AlbB-infected WB1 mosquitoes relative to the uninfected Waco line at 7 days old, prior to blood feeding. Each treatment had four biological replicates, with ten midguts or carcasses pooled as one sample. 2^−ΔΔCT^ method was used to calculate the fold change for each gene. Significance was determined based on comparison of the ΔCT of each gene in WB1 and Waco using Student’s *t*-tests. *P<0.05; **P<0.01. Survival curves of the mosquitoes post-challenge with *E. cloacae* (**c**), *M. luteus* (**d**) or *B. bassiana* (**e**). WB1 is an *A. aegypti* line carrying a stable *w*AlbB infection. WBT is an aposymbiotic line generated by removing *w*AlbB from WB1 by tetracycline treatment, and Waco is a wild-type *Wolbachia*-uninfected line. Each treatment had either six (**c** and **d**) or three (**e**) biological replicates of 15–20 mosquitoes each. Error bars indicate the standard error. Survival curves are significantly different between WB1 and other groups (compared using log-rank test).

We have previously shown that *w*AlbB infection activates the Toll pathway in *A. aegypti* (Pan *et al.*, 2012). To determine a possible effect of *w*AlbB on the IMD pathway, we conducted quantitative reverse-transcription polymerase chain reaction (qRT-PCR) assays comparing the transcript abundance of 13 immune genes from the IMD and Toll pathways. Total RNA was prepared from midguts and the remaining carcass tissues of 7-day-old female mosquitoes of the WB1 and wild-type Waco strains. Consistent with the previous report (Pan *et al.*, 2012), transcripts of three assayed Toll pathway genes—GNBPB1, SPZ3B and MYD88—were strongly induced in both the midguts (6.4-, 5.5- and 2.7-fold, respectively; *P*<0.05) and carcass tissues (3.3-, 3.8- and 2.7-fold, respectively; *P*<0.01) (Figures 1a and b). In addition, REL1 was induced 4.3-fold by *w*AlbB in the carcass (*P*<0.05) (Figure 1b). The transcripts of two assayed IMD pathway genes—peptidoglycan recognition protein (PGRP)-LE, and REL2—were elevated greater than twofold in the carcasses of WB1 when compared with Waco mosquitoes (*P*<0.01) (Figure 1b); in the midguts of WB1, PGRP-LE, PGRP-LB, IMD and REL2 were induced about 2-fold, although not significantly. DEFA was induced >22-fold in the midgut (*P*<0.05) of WB1 mosquitoes, and CECA and CECD were induced 7.4-fold and 7.7-fold in midguts and carcass (*P*<0.05), respectively (Figures 1a and b). These results suggest that the simultaneous induction of Toll and IMD in WB1 mosquitoes may result in an enhanced synergistic effect on the expression of effector molecules, which is consistent with our previous report (Zou *et al.*, 2011).

To test whether the *w*AlbB-based immunity boost in female mosquitoes affects the susceptibility of these mosquitoes to pathogens, we compared their survivorship after challenge with Gram-negative bacteria (*Enterobacter cloacae*), Gram-positive bacteria (*Micrococcus luteus*) or fungi (*Beauveria bassiana*) with that of the Waco wild-type strain and aposymbiotic WBT strain (generated by tetracycline treatment of WB1 mosquitoes to remove *w*AlbB). Consistent with IMD pathway induction, WB1 mosquitoes survived significantly better than both Waco (*P*<0.01) and WBT (*P*<0.001) after challenge with *E. cloacae* (Figure 1c). Activation of the Toll pathway is expected to protect mosquitoes from Gram-positive bacterial and fungal infections. Indeed, WB1 mosquitoes had a higher survival rate than Waco and WBT when challenged with either *M. luteus* (*P*<0.001) or *B. bassiana* (*P*<0.05) (Figures 1d and e).

### Suppression of the IMD pathway reduces the levels of *w*AlbB in both *A. aegypti* cells and mosquitoes

**Figure 2:**
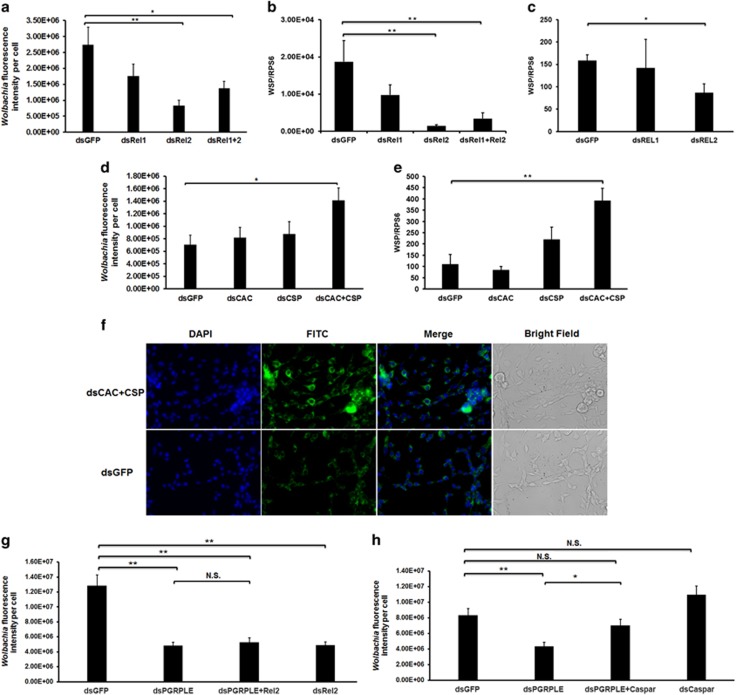
*w*AlbB load changes in response to manipulation of the Toll and IMD pathways via RNAi-mediated gene silencing. The amount of *w*AlbB was measured using either IFA (**a**) or real-time PCR (**b** and **c**) in the *A. aegypti* cell W-Aag2 (**a** and **b**) and WB1 mosquitoes (**c**) after treatment with dsRNA of Rel1, Rel2, both or GFP (control). The same measurements were performed again, after the negative regulators of the Toll and IMD pathways, cactus (CAC) and caspar (CSP), respectively, had been silenced either individually or together (CAC+CSP) to activate these pathways in the W-Aag2 cell line (**d** and **e**). Representative IFA images of dsRNA-treated W-Aag2 cells at x20 magnification with *Wolbachia* surface protein (WSP) staining (green) (**f**). The *Wolbachia* fluorescence intensity was measured in W-Aag2 cells after PGRP-LE was knocked down, either individually or together with either REL2 (**g**) or Caspar (**h**). Error bars indicate the standard error. *P<0.05; **P<0.01; N.S., no significance; one-way ANOVA.

Because *Wolbachia* is a Gram-negative bacterium, we initially hypothesized that the boosted immunity, in particular the activation of the IMD pathway, might play an important role in preventing the over-proliferation of *w*AlbB in WB1 mosquitoes. To test this suggestion, we knocked down REL1, REL2 or both simultaneously using their respective double-stranded (ds) RNAs in the *w*AlbB-infected *A. aegypti* Aag2 cell line (W-Aag2). *Wolbachia* fluorescence intensity was measured using indirect IFA 3 days post treatment. Surprisingly, the level of *w*AlbB was reduced significantly in cells with either single REL2 silencing or a double knockdown of REL1 and REL2 when compared with the dsGFP-treated control (Figure 2a, Supplementary Figures S1A and B). The experiment was repeated independently, with the density of *w*AlbB measured using RT-PCR assay 5 days post treatment (Figure 2b). Consistently, the copy number of the *w*AlbB genome measured by taking the value of *Wolbachia* surface protein gene normalized to the host RPS6 gene was reduced 13.2- and 5.6-fold after single REL2 and dual REL1/REL2 knockdowns in W-Aag2 cells, respectively (Figure 2b). To exclude the possibility that such reduction was caused by a change in the number of copies of host RPS6, we compared the absolute number of copies of *Wolbachia* surface protein among different treatments. With the silencing of REL2 and co-silencing of both REL1 and REL2, the *Wolbachia* surface protein copy number was significantly lower in W-Aag2 cells than in the control (*P*<0.05) (Supplementary Figure S2). To further confirm this result, we carried out a similar knockdown in WB1 mosquitoes and measured the amount of *Wolbachia* in mosquito ovaries 4 days after treatment. Again, we observed a significant reduction in the *w*AlbB load in WB1 ovaries when REL2 was silenced (Figure 2c). We failed to get results from mosquitoes with the double knockdown of REL1 and REL2 because of a very high mortality in treated mosquitoes. These results indicate that suppression of the IMD pathway reduces *Wolbachia* density in both *A. aegypti* cells and mosquitoes.

### Boosting mosquito immunity increases *w*AlbB load in *A. aegypti* cells

To test whether boosting the Toll and IMD pathways would produce an effect on *Wolbachia* opposite to that observed following suppression of these two pathways, we treated W-Aag2 cells with dsRNA of Cactus and Caspar, the respective negative regulators of the Toll and IMD pathways, to induce the individual pathway (Xi *et al.*, 2008b). Double silencing of both Cactus and Caspar was also conducted to activate both pathways simultaneously. In two independent assays, *w*AlbB density was measured using either IFA or RT-PCR assay 3 or 5 days post treatment, respectively. The results consistently show a significant increase in the *Wolbachia* intensity in the cells following simultaneous silencing of Cactus and Caspar when compared with the dsGFP-treated control (Figures 2d and f; Supplementary Figures S3A and B). This increase was not observed in individual knockdowns of either Cactus or Caspar. These results show that boosting immunity promotes proliferation of *w*AlbB in mosquito cells.

### The IMD pathway intracellular receptor PGRP-LE facilitates *w*AlbB growth in *A. aegypti* cells

PGRP-LE has been shown to function upstream of the IMD pathway, serving as the only known intracellular PRR of Gram-negative bacteria in Drosophila (Kaneko *et al.*, 2006; Waterhouse *et al.*, 2007; Yano *et al.*, 2008). Because the expression of PGRP-LE was induced by *w*AlbB in the carcass of *A. aegypti* (Figure 1b), we investigated whether PGRP-LE might have a role in sensing *Wolbachia* in the IMD pathway to facilitate *w*AlbB growth in *A. aegypti*. We conducted RNAi silencing of PGRP-LE, REL2 and both, and measured their impact on *w*AlbB density in the W-Aag2 cell relative to that of the control group treated with dsGFP. We observed that *Wolbachia* fluorescent intensity was significantly reduced by 2.6-fold after either PGRP-LE or REL2 was silenced (*P*<0.0001) (Figure 2g, Supplementary Figures S4A and B). Suppression of the IMD pathway by co-silencing both PGRP-LE and REL2 genes caused no further decrease of *Wolbachia* fluorescent intensity compared with the silencing of PGRP-LE alone (Figure 2g). To validate whether the effect of PGRP-LE on *w*AlbB infection level was modulated through the IMD pathway, we further compared *w*AlbB density in W-Aag2 cells after silencing PGRP-LE, Caspar, and both in W-Aag2 cells (Supplementary Figures S4C and D). We observed that, although the *w*AlbB density was reduced after silencing of PGRP-LE, it was restored to a level similar to the control group after double knockdown of PGRP-LE and Caspar (Figure 2h). These results indicate that both PGRP-LE and REL2 influence *w*AlbB through the IMD pathway, and PGRP-LE may serve as a PRR to sense *Wolbachia* upstream of the IMD pathway.

### Boosting mosquito immunity increases *w*AlbB load in transinfected *A. aegypti*

**Figure 3:**
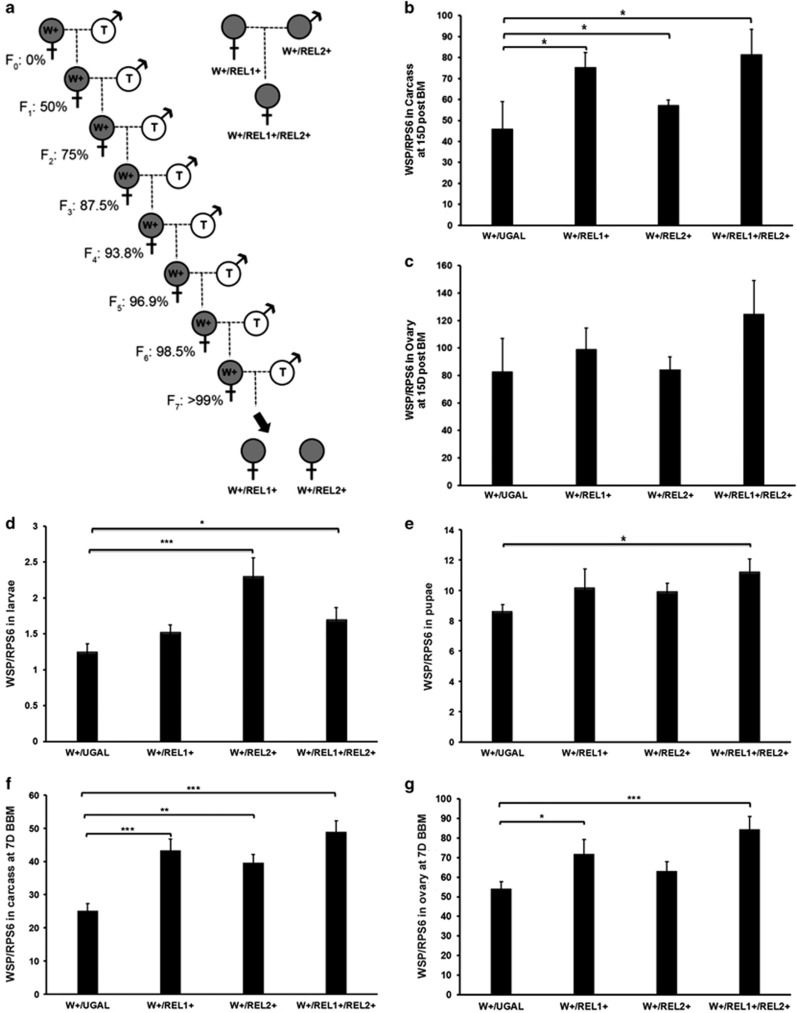
Boosted basal immunity promotes the growth of *w*AlbB in *A. aegypti*. An introgression strategy was used to generate *w*AlbB infection in genetically modified *A. aegypti* with REL1, REL2 or both overexpressed (**a**). *w*AlbB infection is indicated by shading of the symbols: gray-filled with ‘W+’ represents *w*AlbB infection from the WB1 mosquito line; unshaded with ‘T’ represents transgenic *A. aegypti* mosquitoes (REL1+ or REL2+ line). Although the *Wolbachia* infection is maternally inherited, the genetic background is inherited from both parents. The theoretical percentage of transgenic mosquito genetic background is shown below the symbols. Repeated crosses of *Wolbachia*-infected females with transgenic males results in the *Wolbachia*-infected transgenic mosquito lines, W+/REL1+ and W+/REL2+. W+/Ugal was generated as a control by crossing WB1 females with the wild-type Ugal strain in parallel. The *w*AlbB-infected hybrid line, W+/REL1+/REL2+, was generated based on a single cross of W+/REL1+ (G_6_) female and REL2+ male mosquitoes. The copy number of *w*AlbB in the carcass tissues (**b**) and ovaries (**c**) of the three transgenic mosquito lines (W+/REL1+, W+/REL2+ and W+/REL1+/REL2+) was compared with the W+/UGAL control group at day 15 post-blood feeding (PBM) using real-time PCR. The amount of wAlbB in the third instar larvae (**d**), pupae (**e**) and adults (**f** and **g**) of the three transgenic lines was further measured using q-PCR and compared with the control group (W+/UGAL). For the adults, the *Wolbachia* density was measured in carcasses (**f**) and ovaries (**g**) of females at 7 days before blood meal (BBM). Each group contained eight biological replicates, each with an individual tissue. Error bars indicate standard error. Asterisks indicate significant difference of *Wolbachia* density in the transgenic lines compared with the control group. *P<0.05; **P<0.01; ***P<0.001; one-way ANOVA.

Manipulating the immune pathway by mean of dsRNA-mediating gene silencing produces only a transient effect. To further test the effect of boosting the Toll and IMD pathways on *Wolbachia* in *A. aegypti*, we took advantage of transgenic lines that overexpress REL1 (REL1+) and REL2 (REL2+), and in which these immune regulatory factors are activated via the blood-meal inducible Vitellogenin (Vg) promoter (Bian *et al.*, 2005; Antonova *et al.*, 2009). *w*AlbB was introduced into REL1+ and REL2+ mosquito lines through repeated crosses of *w*AlbB-infected females with REL1+ and REL2+ transgenic males for seven generations (Bian *et al.*, 2005; Antonova *et al.*, 2009). These crosses resulted in *w*AlbB-infected transgenic mosquitoes, referred to as W+/REL1+ and W+/REL2+. A cross of *w*AbB-infected mosquitoes with wild-type UGAL/Rockefeller strain mosquito, from which the transgenic lines were derived, was used to generate a control line, W+/UGAL. A single cross between W+/REL1 and W+/REL2+ was used to generate a hybrid line with co-activated Toll and IMD pathways, referred to as W+/REL1+/REL2+ (Figure 3a). To validate the activation of the transgenes in these lines, we measured the expression of REL1 and REL2 in mosquito fat bodies 7 days post eclosion and 24 h post blood meal (PBM). Consistent with previous reports (Bian *et al.*, 2005; Antonova *et al.*, 2009), the expression of the transgenes did not change before a blood meal, but were induced markedly at 24 h post blood meal (Supplementary Figure S5), indicating that *Wolbachia* did not change the regulation patterns of transgene expression. We then compared *w*AlbB load in both the ovaries and the remaining carcass tissue of these mosquitoes at day 15 post blood meal. *Wolbachia* load was significantly higher in the carcasses of W+/ REL1+, W+/REL2+ and W+/REL1+/REL2+ mosquitoes than in W+/UGAL mosquitoes (Figure 3b). No similar increase was observed in the ovaries (Figure 3c). Taken together, these results show that boosting immunity promotes proliferation of *w*AlbB in *A. aegypti.*

### Maternal transmission of elevated *Wolbachia* densities in *A. aegypti* mosquitoes with enhanced immunity background

To test whether the elevated *w*AlbB infection could be maternally transmitted to the offspring of *A. aegypti* mosquitoes with a transgenically enhanced immunity background, we measured the *w*AlbB load in these offspring at the third instar larval and pupal stages. Higher *w*AlbB load was observed in both W+/REL2+ larvae (*P*<0.01) and two immature stages of the W+/REL1+/REL2+ (*P*<0.05 for the larvae and *P*<0.01 for the pupae) transgenic mosquitoes than in W+/UGAL mosquitoes (Figures 3d and e). We further compared the *w*AlbB load at 7 days post eclosion in adult offspring that had not been blood fed and observed a significantly higher load of *w*AlbB in the carcasses of all three transgenic lines—W+/REL1+, W+/REL2+, and W+/REL1+/REL2+—than in the W+/UGAL line (*P*<0.01) (Figure 3f). A significant increase in *w*AlbB load was also observed in the ovaries of non-blood fed W+/REL1+ and W+/REL1+/REL2+ mosquitoes when compared with W+/UGAL mosquitoes (Figure 3g). These results indicate that an elevated level of *w*AlbB infection is maintained through maternal transmission of the bacteria to the offspring of transgenic *A. aegypti* mosquitoes.

### Antioxidants, as potential effector molecules of boosted immunity, enhance *Wolbachia* infection in *A. aegypti*

**Figure 4:**
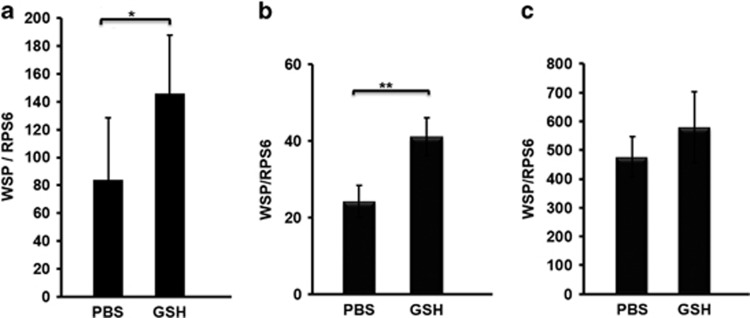
The impact of antioxidant treatment on *Wolbachia* abundance in transfected *A. aegypti* lines. Mosquitoes carrying *w*AlbB (**a**), *w*Mel (**b**) and *w*MelPop (**c**) *Wolbachia* were injected with reduced L-glutathione (GSH) or PBS (control). The genome copy of *Wolbachia* was measured using q-PCR in the mosquito whole body 12 days post treatment. Each treatment had eight biological replicates with one mosquito each. Error bars indicate the standard error. Asterisks indicate significant difference of *Wolbachia* density in the treatment group compared with the control group. *P<0.05; **P<0.01; Mann–Whitney *U*-test.

Overexpression of antimicrobial peptides was reported to increase tolerance to oxidant stress in *Drosophila melanogaster* (Zhao *et al.*, 2011), and both *w*AlbB and boosting mosquito immunity were observed to induce the expression of antioxidants and antimicrobial peptides in *A. aegypti* (Pan *et al.*, 2012). We speculated that boosting immunity enhances *Wolbachia* infection through alterations in the redox balance. To explore whether an antioxidant could act as an effector molecule to regulate *w*AlbB infection levels, we injected WB1 mosquitoes intrathoracically with glutathione and then measured the *w*AlbB levels in mosquito whole bodies 12 days post treatment. We observed a 1.7-fold increase in the *w*AlbB load in WB1 mosquitoes treated with glutathione compared with those treated with PBS as control (*P*<0.05; Figure 4a). To test whether this regulation occurred across different *Wolbachia* strains in *A. aegypti*, we also did a similar comparison using *A. aegypti* carrying a stable *w*Mel (MGYP2 line) and *w*MelPop (PGYP1 line) (McMeniman *et al.*, 2009; Walker *et al.*, 2011). The glutathione treatment again caused a 1.7-fold increase in the amount of *w*Mel in the MGYP2 mosquito compared with control (*P*<0.01) (Figure 4b). Although there was an increase in the *w*MelPop titer in the PGYP1 line relative to control, the difference was not statistically significant (Figure 4c). These results indicate that antioxidants may serve as the downstream effector molecules that enhance *Wolbachia* infection owing to their ability to counterbalance reactive oxygen species induced by this bacterium.

## Discussion

Activation of the host’s innate immune system by *Wolbachia* has been suggested as one mechanism causing pathogen interference in both naturally infected *Drosophila* and transinfected *A. aegypti* mosquitoes (Moreira *et al.*, 2009; Bian *et al.*, 2010; Rances *et al.*, 2012; Wong *et al.*, 2015), but the impact on *Wolbachia* itself has remained unclear. In this work, we have shown that both the IMD and Toll pathways in *A. aegypti* are triggered by introduction of the bacterium *Wolbachia w*AlbB. Enhancing these pathways causes an increase in the *w*AlbB titer, whereas silencing results in a reduction. Moreover, these elevated *w*AlbB infection levels are maintained through maternal transmission. We have also shown that antioxidants might serve as downstream effector molecules of the host immune system that enhance *Wolbachia* infection. Thus, our study has revealed the basis of interactions between *Wolbachia* and a newly acquired host.

Our results suggest that *w*AlbB can be sensed by the mosquito’s innate immune system via PGRP-LE as a PRR, resulting in activation of the IMD pathway. This is consistent with the observation that PGRP-LE is an intracellular sensor of Gram-negative bacteria that induces the IMD pathway in *Drosophila* (Kaneko *et al.*, 2006). It would be interesting to know whether PGRP-LE detects *Wolbachia* through its DAP-type peptidoglycan. Remarkably, boosted immunity markedly induces the expression of effector molecules that do not inhibit, but rather promote the proliferation of *w*AlbB in *A. aegypti*. This is most likely due to a lack of any specific targets for those antimicrobial peptides in the *Wolbachia* cell membrane. An increase in *Wolbachia* density was also reported in tsetse flies following induction of antimicrobial peptides by trypanosome infection (Rio *et al.*, 2006). We think that an increase in *Wolbachia* density following immune boosting could be due to production of effector molecules that facilitate *Wolbachia* growth. One example could be antioxidants, the expression of which was previously shown to be induced or suppressed by activation or silencing, respectively, of the Toll pathway in *A. aegypti* (Pan *et al.*, 2012). In *Drosophila*, overexpression of antimicrobial peptides increases antioxidant enzyme activities and changes the cellular redox balance, facilitating fly survival in hyperoxia (Zhao *et al.*, 2011). Consistent with our observation in both WB1 and MGYP2 mosquitoes, previous studies also showed that antioxidant treatment resulted in increased *Wolbachia* density in *Drosophila* (Brennan *et al.*, 2012). It is also possible that these induced antimicrobial peptides provide an indirect benefit to *Wolbachia* by removing the susceptible microbial flora from within mosquitoes and generating new niches. As a consequence, *Wolbachia* may utilize the immune boost to exclude its competitors and take their place in various tissues. Taken together, *Wolbachia* activates mosquito immune pathways, which leads to promotion of its own growth, indicating that there is a positive feedback loop between the host immune system and *Wolbachia* load. In transinfected *A. aegypti* lines, the observed immune system boost provides survival signals for successful establishment of a novel *Wolbachia* symbiosis.

Previous studies have also shown that the host immune system interacts with symbiotic bacteria to foster relationships in other systems. In *D. melanogaster*, the endosymbiont *Spiroplasma* is not susceptible to either the cellular or humoral arms of the host immune system, and activation of the Toll and IMD immune pathways results in an increase in the *Spiroplasma* titer (Herren and Lemaitre, 2011). Evidence from both invertebrate and vertebrate models reveals that innate immune receptors are required to promote long-term colonization by the microbiota (Chu and Mazmanian, 2013). The host immune system has a central role in shaping the composition of the microbiota as well as its proximity to host tissues (Hooper *et al.*, 2012). The immune system of organisms ranging from hydra to mammals can distinguish beneficial from harmful bacteria via PRRs and interact with symbionts in a way that promotes the establishment and maintenance of beneficial symbioses (Nyholm and Graf, 2012). Alternatively, symbiotic bacteria may utilize the host immune system to actively promote highly evolved associations with their hosts (Hooper *et al.*, 2012; Nyholm and Graf, 2012; Chu and Mazmanian, 2013). *Wolbachia* may use a similar strategy to exploit the host immunity to establish symbiosis.

Boosting of both the Toll and IMD pathways was consistent with the function assay through comparison of the survivorship of *w*AlbB-infected and uninfected *A. aegypti* after challenge by Gram-negative bacteria, Gram-positive bacteria and fungi. We observed that *w*AlbB protects WB1 mosquitoes from all three types of pathogen. This is in agreement with a previous report showing that both *w*Mel and *w*MelPop can protect *A. aegypti* from infection with Gram-negative bacteria (*Erwinia* *carotovora* and *Salmonella* *typhimurium*), and *w*MelPop can increase survival of mosquitoes after challenge with Gram-positive bacteria (*Mycobacterium* *marinum*) (Ye *et al.*, 2013). Thus, *Wolbachia-*boosted immunity is beneficial to the mosquito host owing to *Wolbachia*-associated protection against pathogens. In turn, this facilitates establishment of the long-term relationship between *Wolbachia* and its new mosquito host. As defensive symbionts, *Spiroplasma and Hamiltonella defensa* protect their hosts from attack by a nematode and a parasitoid, respectively, promoting spread of symbionts in natural populations (Oliver *et al.*, 2008; Jaenike *et al.*, 2010). *Wolbachia* might use a similar strategy to accelerate and maintain its cytoplasmic incompatibility-driven spread, facilitating the current effort to develop a *Wolbachia*-based population replacement for dengue and Zika control.

The work reported here has three important implications. First, mosquito innate immunity can be finely adjusted such that it inhibits pathogens but promotes endosymbionts. Understanding this mechanism will greatly facilitate the development of endosymbionts as a tool for blocking pathogen transmission. For example, boosting immunity through mosquito transgenesis could be used to enhance both pathogen refractoriness and *Wolbachia* symbiosis simultaneously. Second, the interplay between the mosquito innate immune system and symbiosis can occur in two directions. On the one hand, the mosquito microbiota facilitates the buildup of basal immunity, as removal of the microbiota reduces the level of basal immunity (Xi *et al.*, 2008b; Dong *et al.*, 2009). On the other hand, a high level of basal immunity facilitates the formation of symbioses and promotes the growth of the specific microbes within the mosquito. Finally, the effector molecules induced by mosquito immune pathways, including *Wolbachia*-upregulated antimicrobial peptides such as DEF and CEC, do not inhibit the growth of *Wolbachia*. Future research should investigate how innate immunity can be utilized to shape the microbiota, with regard to its structure, distribution and amount, such that the mosquito physiological environment can be modified to become inhospitable to human pathogens. This could lead to development of novel strategies for blocking the transmission of human pathogens through mosquito vectors.

In addition to the Toll and IMD pathways, it is also worthwhile to explore the role of the JAK/STATB(Janus Kinase-Signal Transducer Activator of Transcription) and JNK (c-Jun N-terminal kinase) pathways in the *Wolbachia*–host symbiotic relationship in the future studies. Induction of the JAK/STAT pathway by *w*Mel has been reported in *A. aegypti* (Terradas *et al.*, 2017). Evidence also shows that the JAK/STAT pathway controls DENV infection in *A. aegypti* (Souza-Neto *et al.*, 2009) and West Nile virus infection in *Culex* mosquitoes (Paradkar *et al.*, 2012). Previous studies also show that *Wolbachia* upregulates genes in the JNK pathway (Xi *et al.*, 2008a; Kremer *et al.*, 2012), and this pathway was reported to confer tolerance to oxidative stress and extend life span in *Drosophila* (Wang *et al.*, 2003). Thus, *Wolbachia* might utilize the host JNK pathway to facilitate its growth through regulation of host oxidative metabolism. It will be interesting to further study how cross-talk between host immune and metabolic pathways facilitates the *Wolbachia*–host communication to establish symbiosis.

Finally, the present study leads to deeper understanding of the evolution of symbiosis and pathogen blocking. We show that *Wolbachia* exploits the host immune system to establish a symbiotic relationship with its new mosquito host. Previous studies have also found that the symbiotic bacterium *Sodalis* induces a strong expression of immunity-related genes that is essential for its ability to persistently infect tsetse flies (Weiss *et al.*, 2008). Boosted immunity promotes proliferation of *Wolbachia,* but inhibits both mosquito and human pathogens. It appears that long-term adaptations between *Wolbachia* and its host may lead to a waning of the *Wolbachia*-induced immune response, resulting in reduction of pathogen blocking and attenuation of *Wolbachia* titer, as observed in *A. albopictus*. It will be worth testing whether loss of *Wolbachia* infection can occur from a break in the positive feedback loop among *Wolbachia*, host immunity, and antioxidants. Our results show that *Wolbachia* in somatic tissue (carcasses) may be more sensitive to immune regulation than in ovaries, indicating that attenuation of pathogen blocking may be more likely to occur than loss of symbiosis. Further understanding of the interplay between *Wolbachia* and host immunity will facilitate a long-term projection of the stability of the *Wolbachia*–*A. aegypti* mosquito system that is being used in the control of *Aedes* mosquitoes and dengue.

## Materials and methods

DNA and RNA extraction, PCR, quantitative reverse-transcription polymerase chain reaction, RNAi, IFA, microbial challenge and survival assays and antioxidant treatment were conducted using standard methods, details of which are available in the supplementary materials and methods.

### Mosquito rearing and cell culture

Waco is a *Wolbachia*-free, wild-type *A. aegypti* mosquito line. WB1 (Xi *et al.*, 2005), *w*Mel (Walker *et al.*, 2011) and *w*MelPop-CLA (McMeniman *et al.*, 2009) are *A. aegypti* mosquito lines that stably carry *Wolbachia w*AlbB, *w*Mel or *w*MelPop infections, respectively. Prior to use, the WB1 line was out-crossed with the Waco line for >10 generations to ensure a similar genomic background. WBT is an *A. aegypti* mosquito line, in which the *w*AlbB infection was cleared by tetracycline treatment of WB1, and has subsequently been maintained for >10 generations. Vg-ΔREL1-A (REL1+) and Vg-REL2 (REL2+) are transgenic *A. aegypti* mosquito lines, in which the Rel1 and Rel2 genes, respectively, are overexpressed (Bian *et al.*, 2005; Antonova *et al.*, 2009). The Rockefeller/UGAL is a wild-type *A. aegypti* line used as a control for the transgenic lines. All mosquito lines used in this study were maintained under standard laboratory conditions of 28 °C and 80% humidity, with a 12- h/12- h light/dark cycle, as previously reported (Pan *et al.*, 2012). This study was carried out in strict accordance with the recommendations set out in the Guide for the Care and Use of Laboratory Animals of the National Institutes of Health. The protocol was approved by Michigan State University Animal Care and Use Committees.

The *w*AlbB-infected W-Aag2 cell line was maintained in Schneider's Drosophila cell culture media supplemented with l-glutamine, 10% fetal bovine serum (v/v) and 1% penicillin/streptomycin (Life Technologies, Carlsbad, CA, USA) at 26 °C following standard procedures described previously (Lu *et al.*, 2012).

### Introduction of *Wolbachia* into transgenic mosquitoes

An introgression strategy was used to generate *w*AlbB-infected *A. aegypti* lines overexpressing REL1(REL1+), REL2 (REL2+) or both (REL1+/REL2+) (Brelsfoard *et al.*, 2008). Transgenic mosquitoes identified by the EGFP eye marker were chosen for this study. In total, 50 WB1 virgin females were crossed with 50 virgin males of each transgenic *A. aegypti* mosquito line REL1+. REL2+ as well as the wild-type Rockefeller/UGAL at 1–2 days post eclosion. The outcross was repeated for seven generations, so that the lines shared over 99% genomic identity with their transgenic counterparts. A hybrid line (W+/REL1+/REL2+) was established through a single outcross between 50 *w*AlbB-infected Rel1+ (W+/REL1+) virgin females (G6) and 50 Rel2+ virgin males. The *w*AlbB infection was stably maintained in the W+/REL1+, W+/REL2+, W+/REL1+/REL2+ and W+/ Ugal mosquito lines throughout the course of the experiment.
