# Supplementary material for: The bacterium Wolbachia exploits host innate immunity to establish a symbiotic relationship with the dengue vector mosquito Aedes aegypti
Source: ISME J. 2017 Nov 3;12(1):277–88. doi: 10.1038/ismej.2017.174 (PMC5739022; doi:10.1038/ismej.2017.174)
Supplement: Supplementary Material and Methods [file ismej2017174x1.docx]

**Supplementary Material and Methods**

**RNA extraction, cDNA synthesis, and qRT-PCR**

Mosquitoes were surface-sterilized in 70 % (v/v) ethanol for 5-10 min before dissection. Total RNA was extracted from homogenized mosquito tissues and cell lines using an RNeasy Mini Kit (QIAGEN), and cDNA was produced using a QuantiTect Reverse Transcription Kit (QIAGEN), as previously described ([Pan et al 2012](#_ENREF_33)). Real-time PCR was performed using a Quantitect SYBR Green PCR Kit (QIAGEN) and an ABI Prism 7900HT Sequence Detection System (Applied Biosystems). The ribosomal protein S6 (RPS6) gene was used to normalize cDNA templates ([Pan et al 2012](#_ENREF_33)). Primers for the GNBPB1, SPZ3B, MYD88, REL1A, PGRP-LB, PGRP-LE, IMD, REL2, ATT, CECD, and DEFC genes have been described previously ([Bian et al 2010](#_ENREF_3), [Pan et al 2012](#_ENREF_33)). Specific primers were designed to amplify the CECA and DEFA genes, and the REL1 and REL2 transgenes for this study (Table S1).

**Genomic DNA extraction and measurement of *w*AlbB densities**

Genomic DNA was isolated from homogenized mosquito tissues and cell samples using a DNAeasy Blood & Tissue Kit (QIAGEN) according to the manufacturer's protocol. The *Wolbachia* load was measured by real-time PCR using specific primers to amplify the *w*AlbB wsp or mosquito RPS6 gene, as described previously ([Lu et al 2012](#_ENREF_25)). Specific primers were designed to amplify *w*Mel wsp for this study (Table S1). Standard curves were generated for absolute copy number determination using a plasmid containing a fragment of the wsp gene from *w*AlbB, *w*Mel, or the mosquito RPS6 gene, as described previously ([Lu et al 2012](#_ENREF_25)). To measure *Wolbachia* density changes post-dsRNA treatment, ovaries and the remaining carcass samples were collected 4 days post-injection, and cell samples were collected 5 days post-transfection for genomic DNA (gDNA) extraction.

**RNA interference-mediated gene silencing**

A T7 Megascript transcription kit (Ambion Inc) was used to synthesize dsRNAs according to the manufacturer’s instructions. The T7 promoter sequence (TAATACGACTCACTATAGGG) was incorporated into both forward and reverse primers, designed to amplify the target genes. The primers used for the dsRel1, dsRel2, dsCactus, dsCaspar, and dsPGRP-LE synthesis have been described previously ([Xi et al 2008b](#_ENREF_49)) and the others used in this study are listed in Table S1. The assay was conducted according to standard methodology ([Dong et al 2006](#_ENREF_12)). Using a nanoinjector, 69ηl of 4µg/µl dsRNA was injected into the thorax of CO_2_-anesthetized, 1- to 2-day-old female WB1 mosquitoes. To evaluate knockdown efficiency, midgut and fat-body samples were collected 3 days post-injection for RNA extraction and expression analysis.

To perform RNAi in W-Aag2 cell line, 1X10^5^ cells were seeded into each well of 24-well plates at 24 h before the transfection. The transfection complexes of 1 μg of dsRNA and Attractene Transfection Reagent (QIAGEN) were added into each well, based on the manufacturer’s protocol. Gene silencing was confirmed at day 3 post-transfection.

**Microbial challenge and survival experiments**

Survival experiments were initiated by challenging mosquitoes with Gram-negative (*Enterobacter cloacae*) and Gram-positive (*Micrococcus luteus*) bacteria, and fungi (*Beauveria bassiana*) ([Bian et al 2005](#_ENREF_2)). In brief, a sterile needle (Hamilton 33S) was dipped into a concentrated overnight bacterial culture, suspension of *Beauveria bassiana* spores (5 × 10^7^ per ml of viable spores), or sterile LB1 culture (negative control) and then injected into the rear part of the abdomen of 1- to 2-day-old females. Each treatment group was comprised of three replicate experiments with 15 mosquitoes per container. The number of dead mosquitoes was recorded on a daily basis. Every week, surviving mosquitoes were transferred to a new container. This procedure continued until all mosquitoes were dead.

**Indirect immunofluorescence assay (IFA)**

At 72 h post-transfection, cells were fixed with 4% paraformaldehyde solution at 4 °C for 1 h, and then incubated with 10% non-fat dry milk blocking solution at room temperature with gentle shaking for 1 h, followed by incubation with a rabbit anti-wsp primary antibody (GenScript) at 1:500 and Alexa flour 488 conjugated secondary antibody (Molecular Probes, Invitrogen) at 1:1000. Subsequently, samples were incubated with 0.1 μg/ml DAPI for 1 min. The fluorescence signal was visualized under an Olympus IX71 microscope. There were three technical replications for each treatment. Four individual images were taken randomly for each well, and the fluorescent intensities were normalized to the cell number in each image, using MetaMorph® Microscopy Automation & Image Analysis Software.

**Effect of GSH on *Wolbachia* density**

*w*AlbB- or *w*Mel-infected *A. aegypti* female mosquitoes were injected with 69ηl of 0.125M reduced L-glutathione (GSH) (Sigma-Aldrich) or 1x PBS (control) 1-2 days PE using a Nano injector. At day 12 post-injection, gDNA was collected from whole-mosquito samples (minus the heads) for *Wolbachia* density detection.

**Statistical analysis**

The survival data from mosquito lines subjected to microbial challenge were analyzed using a log rank test in GraphPad Prism 5. For the data from the IFA assay and real-time PCR assay with more than two experimental groups, the normal distribution and equality of variance were determined first. If variances were not homogeneous, data were subjected to log-10 transformations and one-way analysis of variance (ANOVA) with Dunnett's Multiple Comparison Test using GraphPad Prism 5. A Mann Whitney-U test was used to compare *Wolbachia* densities in mosquitoes after treatment with GSH and PBS. All data are reported as the sample mean ± the standard error.

**Supplementary Figure legends**

**Fig. S1. Knockdown efficiency of Rel1 and Rel2 in W-Aag2 cells.** Data are shown as the relative mRNA levels of Rel1 (A) and Rel 2 (B) after RNAi silencing of either single or both genes in W-Aag2 when compared with dsGFP treatment.

**Fig. S2. The absolute copy number of *w*AlbB in W-Aag2 cells after silencing the Toll and IMD pathway genes by dsRNA.** The copy number of *w*AlbB was measured using real-time PCR in the *A. aegypti* cell W-Aag2 at day 5 post-transfection with dsRNA of Rel1, Rel2, both, or GFP (control). *, *P* < 0.05; one-way ANOVA.

**Fig. S3. Knockdown efficiency of Cactus and Caspar in W-Aag2 cells.** Data are shown as the relative mRNA levels of Cactus (A) and Caspar (B) after RNAi silencing of either single or both genes in W-Aag2 compared with dsGFP treatment.

**Fig. S4. Knockdown efficiency of PGRP-LE, Rel 2 and Caspar in W-Aag2 cells.** Data are shown as the relative mRNA levels of PGRPLE (A and C), Rel 2 (B), and Caspar (D) after RNAi silencing of either single or both genes in W-Aag2 compared with dsGFP treatment.

**Fig. S5. Transgene expression in *Wolbachia*-infected transgenic mosquitoes.** qRT-PCR was used to measure the fold change of Rel1 and Rel2 expression in the fat body of W+/Rel1+, W+/Rel2+, and W+/REL1+/REL2+ lines, relative to W+/Ugal, at 7 days of age, before blood meal (BBM) (A, B) and 24 h post-blood meal (PBM) (C, D). Each treatment consisted of eight biological replicates with an individual tissue each. Asterisks indicate a significant difference. *, *P* < 0.05; **, *P* < 0.01; one-way ANOVA.
