# Supplementary material for: The bacterium Wolbachia exploits host innate immunity to establish a symbiotic relationship with the dengue vector mosquito Aedes aegypti
Source: ISME J. 2017 Nov 3;12(1):277–88. doi: 10.1038/ismej.2017.174 (PMC5739022; doi:10.1038/ismej.2017.174)
Supplement: Supplementary Table S1 [file ismej2017174x2.pdf]

**Table S1. Primer sequences**

| Primer Name | Primer Sequence |
|---|---|
| ds Rel2 forward | 5′-GCTCAGTGCTACCGTGGGAAAC-3′ |
| ds Rel2 reverse | 5′-CGGGTTCGCTCTGGCATTTGTC-3′ |
| ds PGRPLE forward | 5′- CGGTTTCACTGCCTTCCAATGG-3′ |
| ds PGRPLE reverse | 5′- GGCGGTGTGGGATATGATTACG-3′ |
| ds PGRPLB forward | 5′- GAAAAATTTGCCGGACCAATAC-3′ |
| ds PGRPLB reverse | 5′- CCGACGCTCTTATCGTTGTATC-3′ |
| *w*Mel forward | 5′-CCTTTGGAACCCGCTGTGAATG-3′ |
| *w*Mel reverse | 5′-GCCTGCATCAGCAGCCTGTC-3′ |
| CECA forward | 5′-CAGGTGGCCTTAAGAAGCTG -3′ |
| CECA reverse | 5″-GCTTTAGCCCCAGCTACAACT-3′ |
| DFEA forward | 5″-GCCCTTTTGCAAACTCTCTTT-3′ |
| DEFA reverse | 5′-CAATGCAATGAGCAGCACAAG-3′ |
| Rel1 forward | 5′-CATCCAGTGCGTCAAGAAGA-3′ |
| Rel1 reverse | 5′-TCCAGGAAC ACCTGGAAGCA-3′ |
| Rel2 forward | 5′- TGGAAGTGCTCATGCAGCTC-3′ |
| Rel2 reverse | 5′-CGTTGG GAACCACTGTTGGA-3′ |
