# Supplementary figures and images for: The bacterium Wolbachia exploits host innate immunity to establish a symbiotic relationship with the dengue vector mosquito Aedes aegypti
Source: ISME J. 2017 Nov 3;12(1):277–88. doi: 10.1038/ismej.2017.174 (PMC5739022; doi:10.1038/ismej.2017.174)

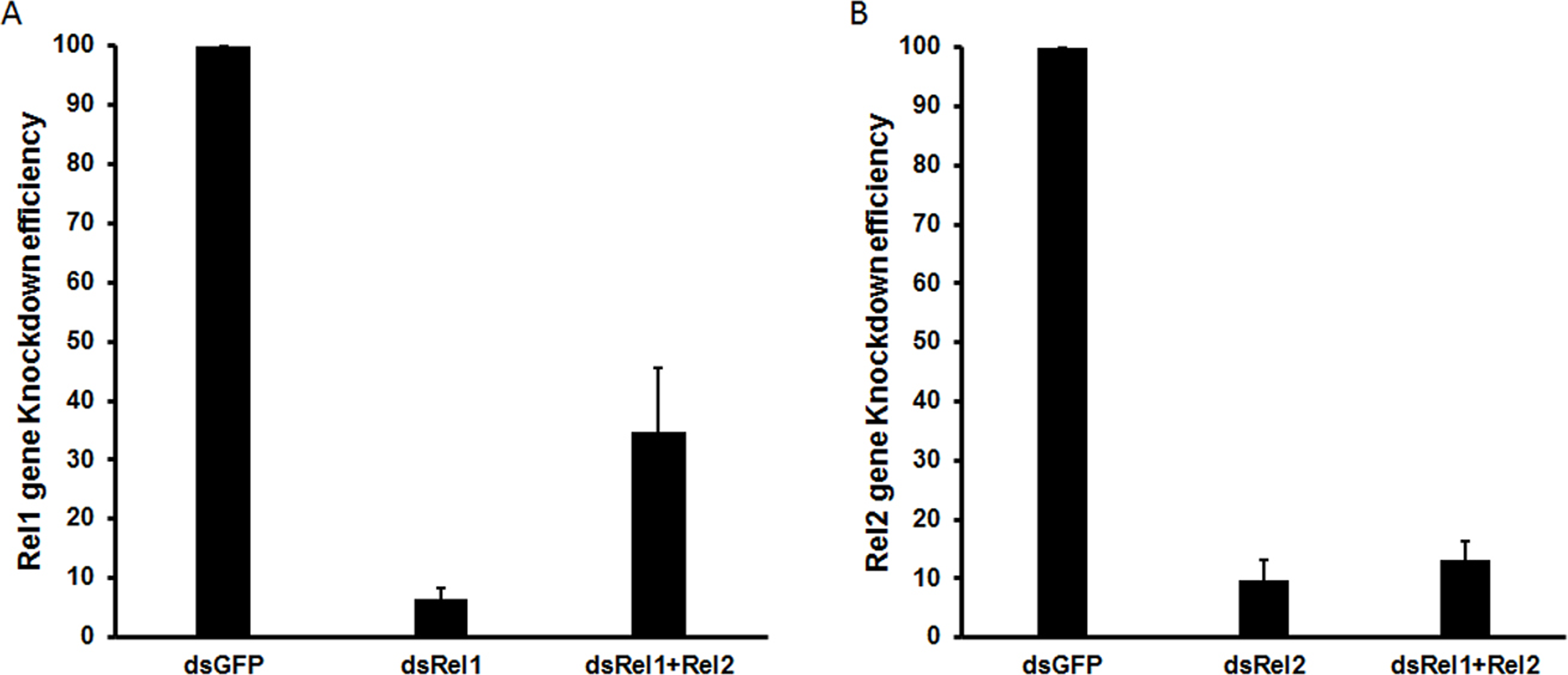

Supplement: Supplementary Figure S1 [file ismej2017174x3.tif]

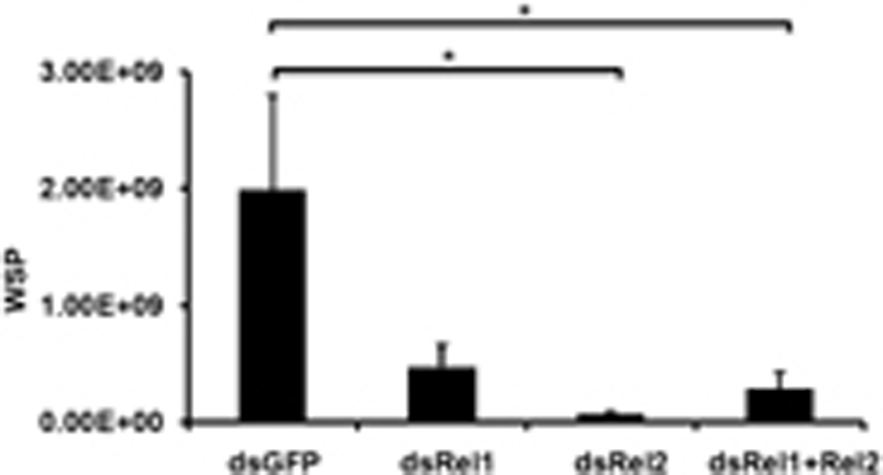

Supplement: Supplementary Figure S2 [file ismej2017174x4.tif]

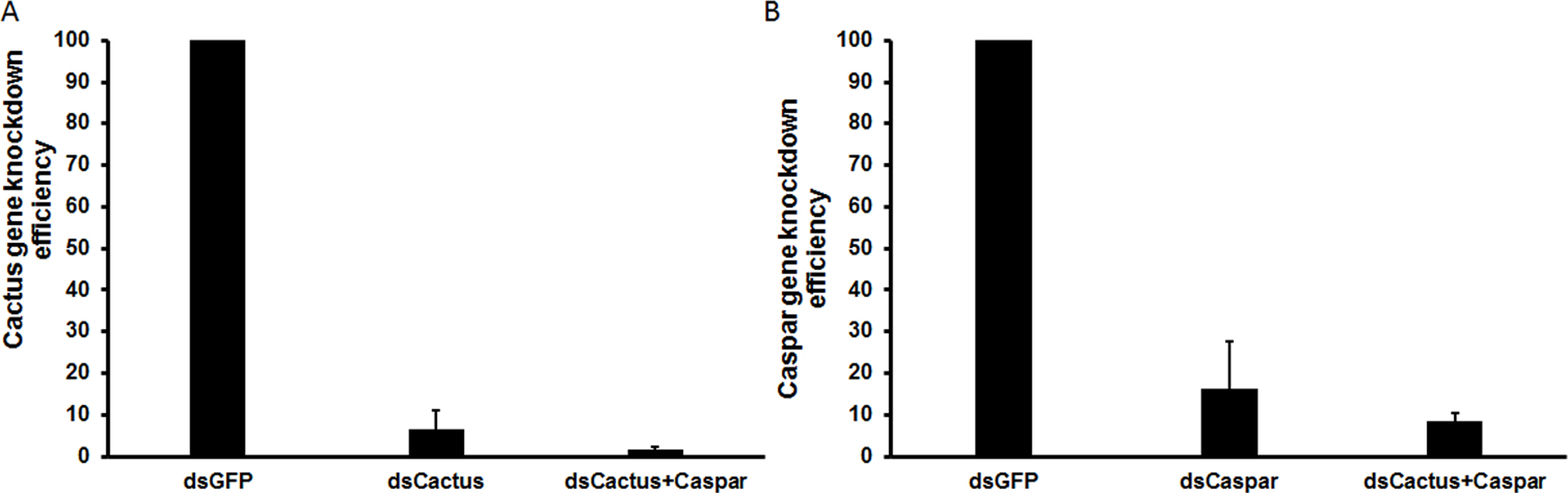

Supplement: Supplementary Figure S3 [file ismej2017174x5.tif]

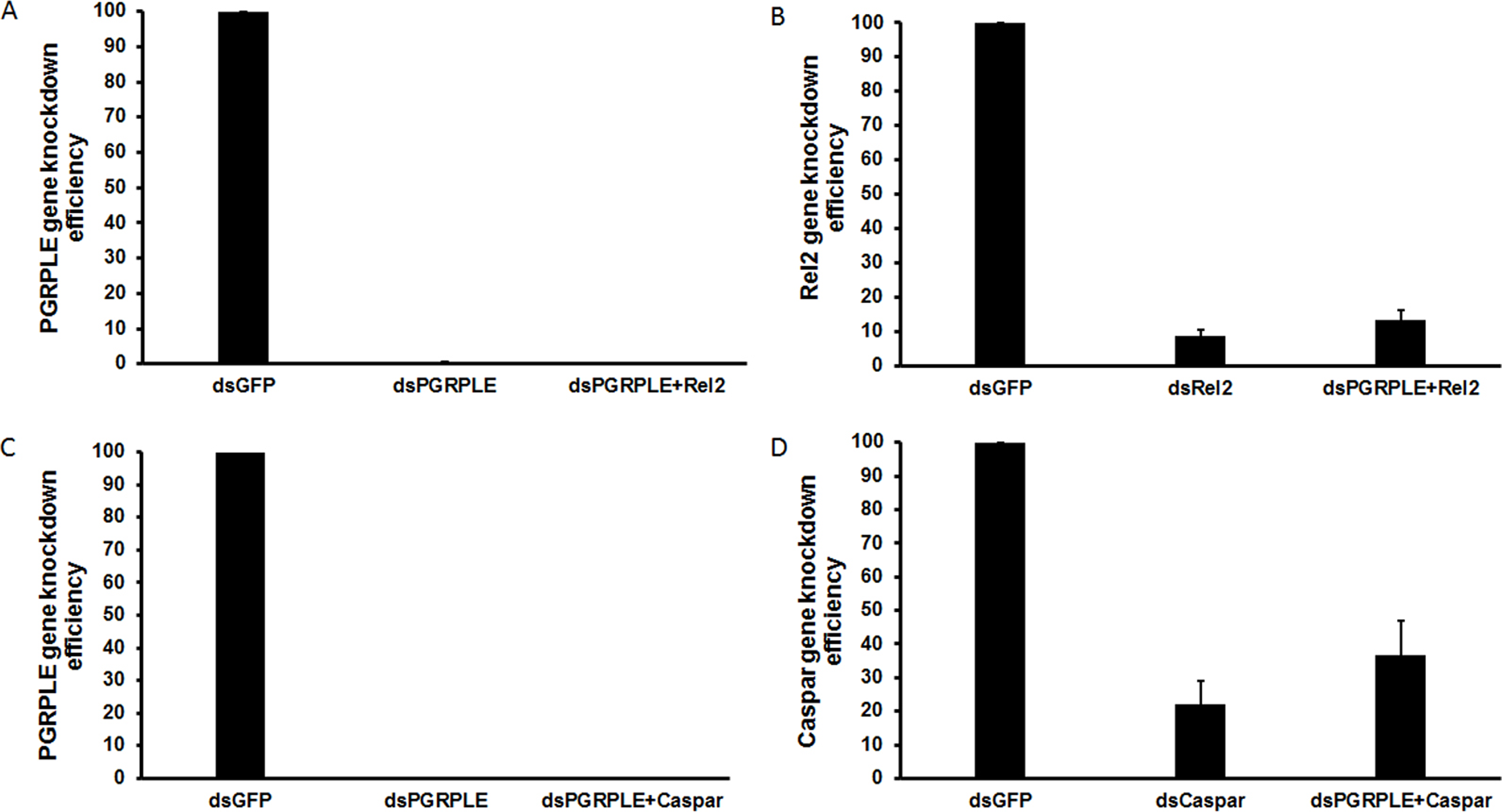

Supplement: Supplementary Figure S4 [file ismej2017174x6.tif]

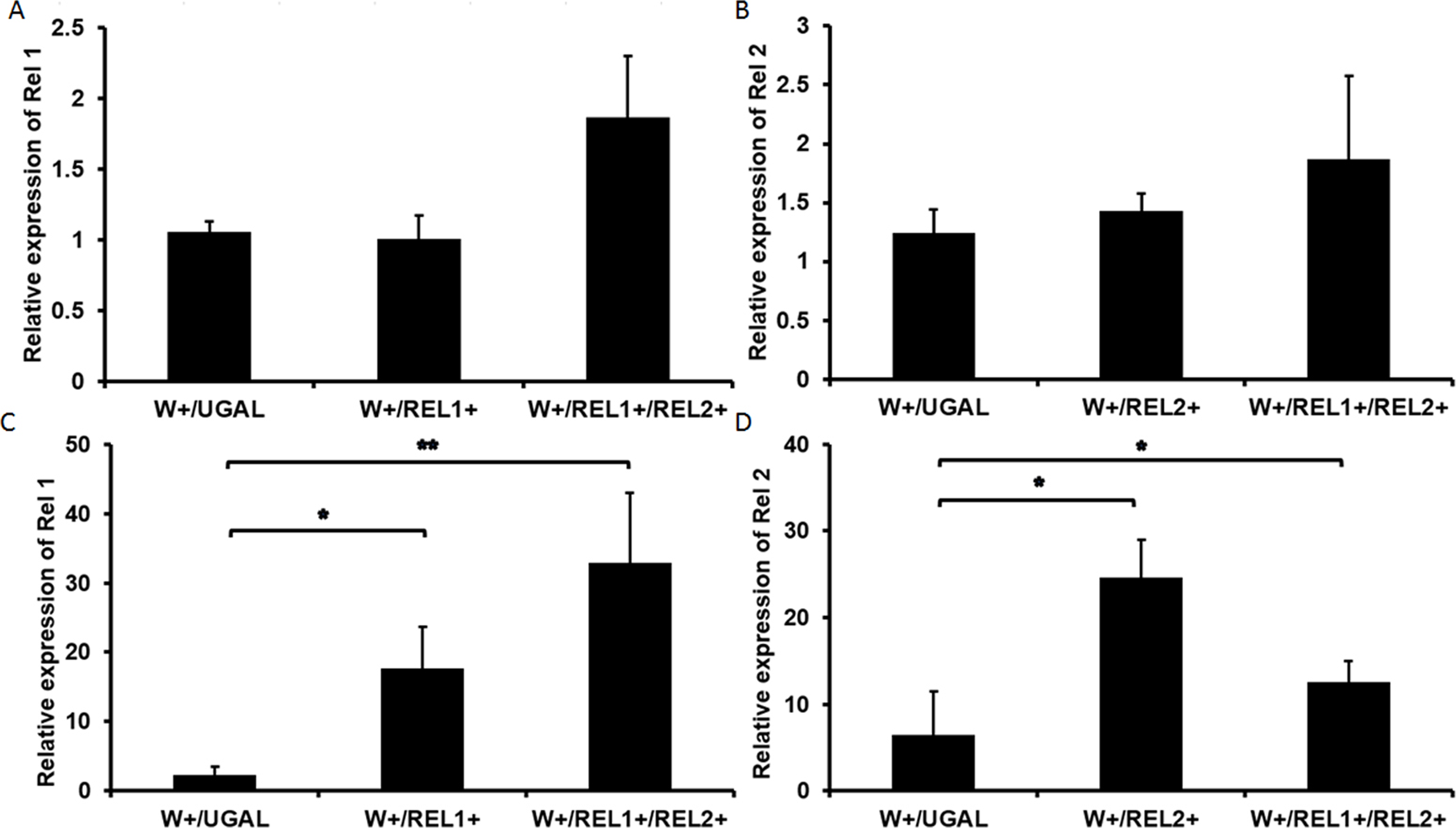

Supplement: Supplementary Figure S5 [file ismej2017174x7.tif]
